# Baiting not-owned dogs against *Echinococcus granulosus*: innovative tools for integrated control

**DOI:** 10.1017/S0031182024000258

**Published:** 2024-04

**Authors:** Elena Ciccone, Antonio Bosco, Paola Pepe, Martina Nocerino, Nicola Lattero, Gerald Umhang, Laatamna AbdElkarim, Samia Lahmar, Yousra Said, Giorgio Saralli, Giuseppe Piegari, Maria Chiara Alterisio, Rania Baka, Smaragda Sotiraki, Franck Boué, Laura Rinaldi

**Affiliations:** 1 Department of Veterinary Medicine and Animal Production, University of Naples Federico II, CREMOPAR, 80137, Naples, Italy; 2 Regional Reference Center for Animal Health (CRESAN), Campania Region, Italy; 3 ANSES, Nancy Laboratory for Rabies and Wildlife Diseases, Technopôle agricole et vétérinaire, BP 40009, 54220 Malzéville, France; 4 Faculty of Nature and Life Sciences, Laboratory of Exploration and Valorization of Steppic Ecosystems, University of Djelfa, Moudjbara Road, BP 3117, Djelfa, Algeria; 5 Parasitology Laboratory, National School of Veterinary Medicine, University of Manouba, 2020 Sidi Thabet, Tunisia; 6 Istituto Zooprofilattico Sperimentale of Lazio and Tuscany M. Aleandri, Via Appia Nuova, 00178 Rome, Italy; 7 Veterinary Research Institute, Hellenic Agricultural Organization-Demeter, 57001 Thessaloniki, Greece

**Keywords:** baits, control programme, *Echinococcus granulosus*, not-owned dogs, praziquantel

## Abstract

Cystic echinococcosis (CE), caused by the larval stage of the cestode *Echinococcus granulosus,* is one of the most widespread zoonoses in Mediterranean countries. Baiting not-owned dogs with praziquantel (PZQ), due to their key role in the maintaining the transmission of CE, currently appears to be the most effective way to limit the transmission of CE, as well as an important aspect to introduce for the control of this parasitic disease. Therefore, this study aims to test 3 types of PZQ-based baits by evaluating different parameters (integrity over time, attractiveness and palatability for dogs, and mechanical resistance after release to different altitudes) and the bait acceptance in field by target animals, i.e. not-owned dogs, by using camera traps. The double PZQ-laced baits (with a double layer of highly palatable chews) showed the greatest resistance in the environment while also preserving the attractiveness and palatability up to 10 days, also withstood heights of 25 m, thus resulting as the most suitable also for drone delivery. The results on the field showed that most of the baits were consumed by not-owned dogs (82.2%), while the remaining were consumed by wild boars (8.9%), foxes (6.7%), badgers (1.1%) and hedgehogs (1.1%), confirming the specific and high attractiveness of the double PZQ-laced baits for the target population and highlights how an anthelmintic baiting programme may be a viable tool for the management of *E. granulosus* among free-ranging dog populations in endemic rural areas.

## Graphical abstract

**Figure:**
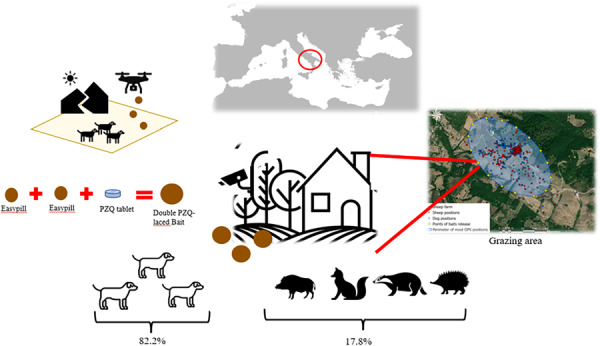

## Introduction

Cystic echinococcosis (CE) is a parasitic zoonosis caused by the larval stage of the tapeworm *Echinococcus granulosus*, a cestode that represents a public health priority due to its worldwide distribution and its impact on human and animal health (Craig *et al*., 2017). CE is now included in the list of the 20 neglected tropical diseases (NTDs) for which control measures are recommended by the World Health Organization (WHO) (WHO, 2011; Casulli *et al*., 2023). The life cycle of the parasite involves a variety of wild and domestic ungulates as intermediate hosts as well as canids as definitive hosts. In pastoral farming of the Mediterranean areas the disease is still highly prevalent in animals and humans, and primarily affects sheep and dogs (Deplazes *et al*., 2017; Cringoli *et al*., 2021). Among European countries, Greece reported an overall prevalence of *E. granulosus* infections in sheep up to 30.4–53.8% (Christodoulopoulos *et al*., 2008; Chaligiannis *et al*., 2015) and 50.4% in shepherd dogs (Sotiraki *et al*., 2003), whereas central-southern and insular regions of Italy reported high values of prevalence up to 75.0% in sheep and 6.0% in dogs (Deplazes *et al*., 2017; Bosco *et al*., 2021; Cringoli *et al*., 2021). The prevalence of CE in Algeria reaches values of 78.0% in sheep (Hamrat *et al*., 2011; Ouchene *et al*., 2014) and 42.0% in dogs (Bentounsi *et al*., 2009), while in Tunisia values of about 40.0% (Lahmar *et al*., 2013) and 21.0% (Lahmar *et al*., 2001) are reported in sheep and in dogs, respectively. Control programmes against *E. granulosus* are considered long-term public health measures that require an integrated approach, including various actions for animals and humans in terms of surveillance, prevention, treatment and education (Craig *et al*., 2017; Cringoli *et al*., 2021). In this framework, new innovative actions to control CE in the Mediterranean areas have been implemented with the aim of increasing surveillance and control strategies against *E. granulosus* in definitive and intermediate hosts (Cringoli *et al*., 2021; Rinaldi *et al*., 2022). However, despite the implementation of such control initiatives, CE still remains a problem in the Mediterranean areas due to its high infection rate.

In this context, the targeted de-worming of dogs with praziquantel (PZQ), i.e. the anthelmintic which has been proven to be effective against mature and immature adult stages of *E. granulosus* (Lightowlers *et al*., 2021), is the mainstay of almost all successful control efforts against CE (Craig *et al*., 2017). Indeed, dogs play a crucial role in the transmission of CE as they can roam freely in grazing areas and prey on livestock, also acting as carriers of others zoonotic diseases (Cringoli *et al*., 2007; Mestel, 2017; Wang *et al*., 2022). These aspects are further exacerbated in the case of not-owned dogs, which are estimated to constitute around 75% of the global dog population (Hughes and MacDonald, 2013) and are considered important reservoirs of *E. granulosus* as well as other zoonotic parasites (Otero-Abad and Torgerson, 2013; Deplazes *et al*., 2017; Otranto *et al*., 2017). Therefore, the potential role of not-owned dogs in contaminating the environment by shedding worms or eggs is a critical issue in the control of CE and other zoonoses (FAO, 2014). Also, the lack of preventive strategies against not-owned dogs, due to their unrestricted freedom of movements, could contribute to a significant increase in the incidence of CE in endemic areas (Deplazes *et al*., 2011; Esch and Petersen, 2013; Baneth *et al*., 2016).

The control of *E. granulosus* infection will necessarily include both owned and not-owned dogs (Kachani and Heath, 2014), so an effective control strategy based on the anthelmintic treatment of not-owned dogs is required to be implemented. An attempt might be to reduce the prevalence of *E. granulosus* by regularly baiting with PZQ, which appears to be the most effective tool for limiting the transmission of CE in dog populations (Lightowlers *et al*., 2021). A similar strategy has been adopted against *E. multilocularis* in fox populations (Eckert and Deplazes, 2004), as PZQ-laced baits released into the environment that are attractive to the target animal species have already been successfully used in several parts of the world in anthelmintic campaigns targeting foxes against *E. multilocularis* (reviewed in Umhang *et al*., 2019), as well as in canids to control *E. granulosus* (Yu *et al*., 2017). However, to date, the use of baits containing PZQ has never been considered as a new control action against CE in Mediterranean countries.

The effectiveness of these strategies requires baits that are attractive and accepted by the target population. To this end, 3 different types of PZQ-laced baits were tested in this study. Different characteristics, such as integrity over time, attractiveness and palatability to the dogs, as well as mechanical resistance at different heights when dropped by a drone were evaluated in order to establish which was most suitable for deworming not-owned dogs, with the aim of implementing CE control programmes in the Mediterranean area. Finally, the acceptability and uptake of the baits by the target animals, i.e. not-owned dogs, was evaluated under field conditions in southern Italy by means of camera traps.

## Materials and methods

### Study design

**Figure 1.**
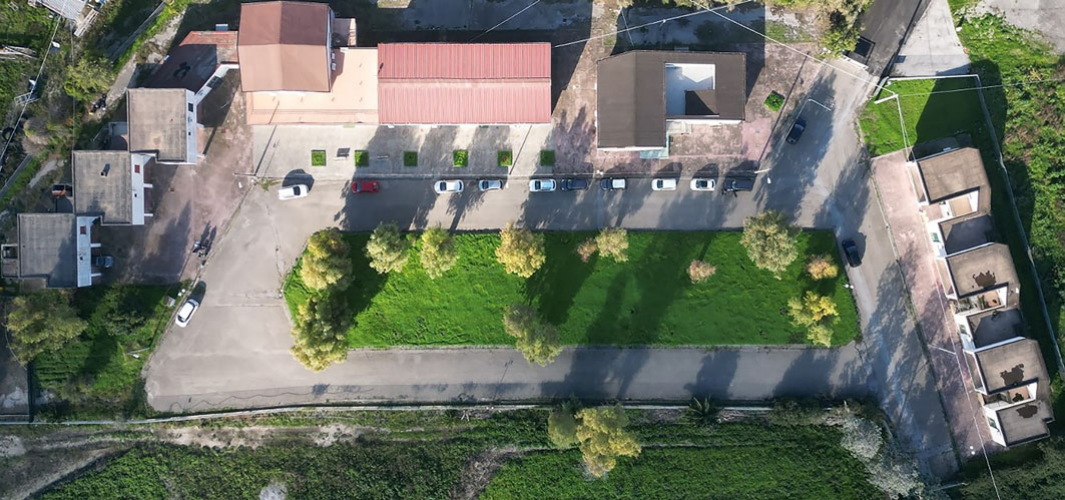
Aerial photograph of the fenced area located within the Center for Monitoring Parasitic Diseases (CREMOPAR, Campania region, southern Italy), characterized by the presence of lawn and trees.

The study was conducted in a pilot area of the Campania region, southern Italy, where CE is highly endemic (Deplazes *et al*., 2017; Bosco *et al*., 2021; Cringoli *et al*., 2021) and was divided into 2 phases. The activities of the first phase, i.e. the *preliminary trial*, were conducted in a delimited and fenced area with lawn and trees (1330 m^2^) located within the Center for Monitoring Parasitic Diseases (CREMOPAR, Campania region, southern Italy) of the Department of Veterinary Medicine and Animal Production, University of Naples Federico II (Fig. 1).

The second phase, i.e. the *field trial on bait acceptability*, was conducted in a grazing area in the Salerno province (Campania region, southern Italy), which is highly endemic for CE, where not-owned dogs and Mediterranean wildlife (e.g. badgers, foxes, hares, hedgehogs, martens, porcupines, roe deer, wild boars, wolves) are widespread.

### Preliminary trial

The preliminary trial was conducted from May 2022 to February 2023. Three different types of baits, consisting of commercially available products with high palatability for dogs, were selected and tested as described below.

Anthelmintic tablets for dogs (weighing between 5 and 25 kg), containing PZQ 125 mg and milbemycin oxime 12.5 mg (Milbemax^®^ tablets – Elanco Italia S.p.A), were packaged in commercially available, highly palatable chews (Giver Dog, EasyPill – Vetinnov, France), to prepare 2 different types of baits:

- the single PZQ-laced baits (consisting of a single layer of Easypill incorporating 1 tablet of Milbemax^®^)
- the double PZQ-laced baits (consisting of a double layer of Easypill incorporating 1 tablet of Milbemax^®^).

A third type of bait (PZQ chewable tablets) was the commercial palatable chew containing PZQ 125 mg and milbemycin oxime 12.5 mg (Milbemax^®^ chew – Elanco Italia S.p.A).

Three parameters were evaluated for each bait: (*i*) integrity over time; (*ii*) attractiveness and palatability for the dogs and (*iii*) mechanical resistance to different altitudes.

#### Integrity over time

**Figure 2.**
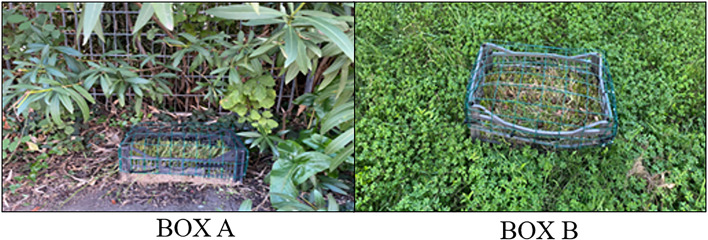
Box A (covered under a bush) and Box B (exposed to sunlight) used to assess the integrity of the baits over time.

To assess the integrity of the baits over time, 2 boxes containing 30 baits of each type were prepared. One box (Box A) was placed on clods of soil and vegetation and covered under a bush; the second box (Box B) was placed in an open field exposed to sunlight (Fig. 2).

The integrity of the baits in the 2 boxes (A and B) was assessed by means of a visual inspection after 5 and 10 days, assigning a score to each bait on a scale of 1–3 (i.e. 1: intact bait; 2: broken bait; 3: mouldy and degenerated bait). The test was carried out in 3 different seasons (spring, summer and winter) to assess the integrity of the baits under different climatic conditions. A total of 540 baits were tested.

#### Attractiveness and palatability for dogs

The attractiveness and palatability of the baits were tested out of a total of 24 owned dogs of different breed, sex and age. The recruited dogs had been free from the anthelminthic treatment for at least 6 months. They were not potentially infected by *E. granulosus* and had been voluntarily included in the study by their owners by signing an informed consent. The intact baits (score 1), selected from either box A or box B, were randomly distributed across the fenced area, with the attractiveness and palatability for the dogs being assessed after both 5 and 10 days. To determine voluntary consumption, each dog was given the opportunity to choose 1 of the 3 bait types. According to Schmid *et al*. (2010) palatability was calculated as follows:

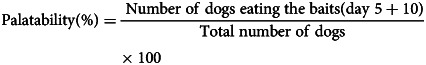


#### Mechanical resistance at different altitudes

A test was conducted to evaluate the mechanical resistance of the 3 types of baits (single PZQ-laced baits, double PZQ-laced baits and PZQ chewable tablets) after release from different heights. To this end, a custom unmanned aerial vehicle (UAV) was used, consisting of a DJI Flame wheel F550 drone equipped with a mechanical part (a dispenser, consisting of 9 wells), which was 3D printed and attached to the frame of the drone (Caputo *et al*., 2022). A total of 135 baits (45 for each type of bait) were released from different heights (5–10–15–20 m) up to the maximum allowable flight altitude (25 m), in locations with different types of ground (soil, vegetation, asphalt), which was followed by a visual inspection. The trial was considered successful if the baits tested had remained completely intact after impact on the ground.

### Field trial on bait acceptability

The trial was conducted from February 2023 to June 2023 in order to have different climatic conditions in the field. Based on the results of the preliminary trial, the double PZQ-laced baits were used to treat not-owned dogs or other canids present in the pilot area of southern Italy in order to evaluate the acceptability by the target species (i.e. not-owned dogs) in the field.

**Figure 3.**
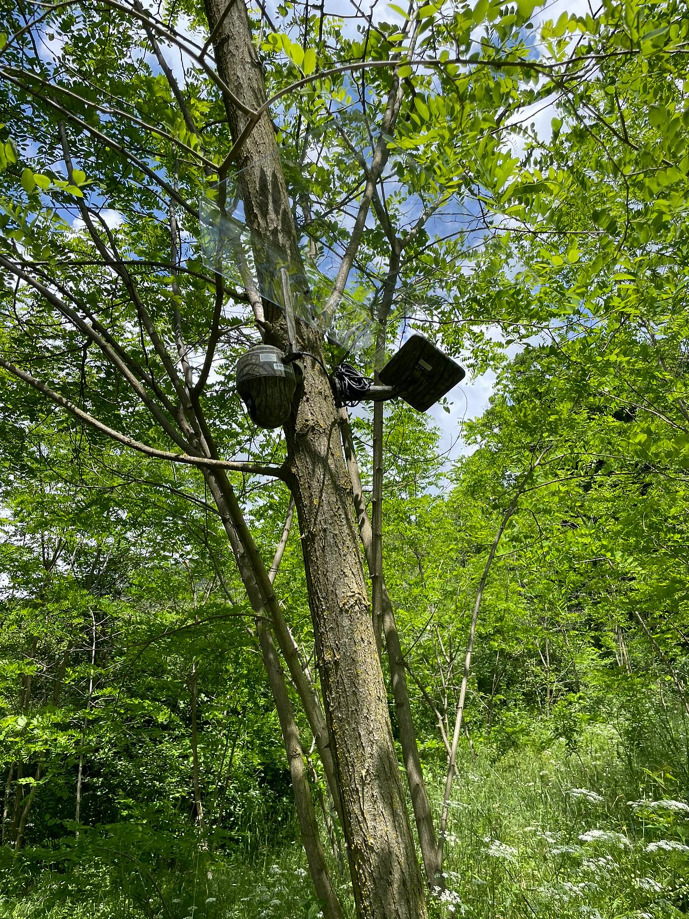
Camera trap installed in the field in order to assess bait acceptance by not-owned dogs.

The area was identified by using mobile global positioning system (GPS) devices (Qtrack GPS, 4G LTE Iot network technology, Austria) applied to sheep and shepherd dogs on a sheep farm that had tested positive for CE. The pilot site for bait delivery was selected by tracking the movements of sheep and dogs, thus identifying some key points (Nocerino *et al*., 2024). The entire pilot baiting site was then divided into units containing 10 fixed points for bait delivery. For each point of release, 3 double PZQ-laced baits were manually placed and replaced every 10 days for the duration of the study. Two camera traps (Reolink KEEN Ranger P, Hong Kong) (Fig. 3) were placed simultaneously at 2 different points of bait release, at a distance of approximately 3 m from the baits, in order to assess bait acceptance by not-owned dogs or other animal species. The position of the 2 camera traps was changed every month until all points selected for bait release were covered.

### Statistical analysis

In the preliminary trial, the correlation between bait types (Single PZQ-laced baits, Double PZQ-laced baits and PZQ chewable tablets) and weather resistance scores was evaluated using the Pearson's *χ*^2^ test in order to assess the integrity of the baits over time. The analysis was performed separately for each box, day and season.

Differences in bait acceptance by dogs related to gender, age and breed were compared by using the Pearson's *χ*^2^ test.

All statistical analyses were performed using SPSS^®^ Statistics Software (v.26, IBM, Armonk, NY, USA), and a significant level of *P* < 0.05 was used.

## Results

### Preliminary trial

#### Integrity over time

**Figure 4.**
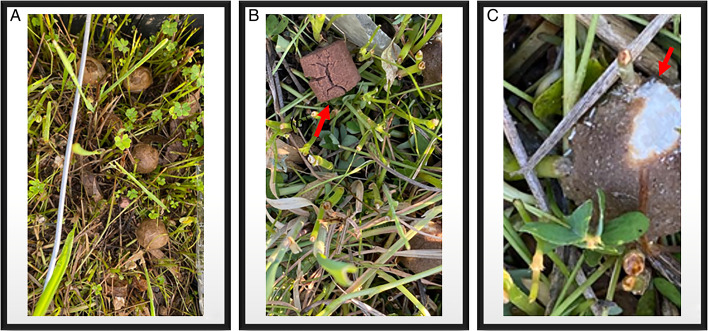
(A) Double PZQ-laced baits at 10 days with score 1 (intact baits); (B) PZQ chewable tablet at 5 days with score 2 (broken bait- see red arrow); (C) Single PZQ-laced bait at 5 days with score 3 (degenerated and mouldy bait- see red arrow).

The double PZQ-laced baits showed the greatest resistance in the environment under different climatic conditions, both those covered in clods of soil and vegetation (Box A) and those in an open field and exposed to sunlight (Box B), exhibiting less degradation and better conservation compared to the other types, which were already broken or degenerated from the fifth day (Fig. 4), as reported in Table 1.

**Table 1.** Numbers of baits in box A and box B with different scores: 1(intact bait); 2 (broken bait); 3 (mouldy and degenerated bait), on different days and in different seasons

Box	Type of baits	Weather resistance (No. baits for each evaluation score)																	
		Day 5									Day 10								
		Spring			Summer			Winter			Spring			Summer			Winter		
		Score 1	Score 2	Score 3	Score 1	Score 2	Score 3	Score 1	Score 2	Score 3	Score 1	Score 2	Score 3	Score 1	Score 2	Score 3	Score 1	Score 2	Score 3
**A**	**Single PZQ-laced**	5	5	5	5	6	4	6	4	5	4	6	5	3	6	6	4	6	5
	**Double PZQ-laced**	10	3	2	11	2	2	11	3	1	9	4	2	10	3	2	10	4	1
	**PZQ chewable**	2	6	7	3	6	6	3	5	7	1	7	7	1	5	9	2	5	8
**B**	**Single PZQ-laced**	5	4	6	4	6	5	5	6	4	3	6	6	4	6	5	3	5	7
	**Double PZQ-laced**	12	2	1	11	3	1	11	2	2	10	3	2	10	3	2	9	4	2
	**PZQ chewable**	3	5	7	2	6	7	2	6	7	2	7	6	1	6	8	1	6	8

The difference in integrity over time between the 3 different types of baits was statistically significant (*P* < 0.05).

#### Attractiveness and palatability for dogs

The results showed that the 24 owned dogs included in the study were more attracted to the double PZQ-laced baits compared to the other types of baits (Table 2). The PZQ chewable tablets, on the other hand, resulted to be the least attractive compared to the others. The spontaneous acceptance rate (palatability %) of the double PZQ-laced baits (70.8%) was higher than that of the other bite types (25.0% for the single PZQ-laced baits and 4.2% for the PZQ chewable tablets). Dogs' age, gender and size were found to have no statistically significant impact on bait acceptance (*P* = 0.11).

**Table 2.** Attractiveness and palatability (%) of the baits over time

Type of baits	Composition	Attractiveness over time (No. Dogs (%) that found and consumed the baits)		
		Day 5	Day 10	Palatability (%)
Single PZQ-laced	Single layer of Easypill incorporating 1 tablet of Milbemax(containing praziquantel 125 mg and milbemycin oxime 12.5 mg)	4 (33.3%)	2 (16.7%)	25.0
Double PZQ-laced	Double layer of Easypill incorporating 1 tablet of Milbemax (containing praziquantel 125 mg and milbemycin oxime 12.5 mg)	7 (58.3%)	10 (83.3%)	70.8
PZQ chewable	Chew containing praziquantel 125 mg and milbemycin oxime 12.5 mg	1 (8.4%)	0 (0%)	4.2

#### Mechanical resistance after dropping at different altitudes

All the 45 double PZQ-laced baits (100%) tested remained completely intact after an impact on different surfaces and on any type of ground up to a height of 25 m compared to the other types of baits. Indeed, only 40 PZQ chewable tablets on 45 (90%) and 36 Single PZQ-laced baits on 45 (80%) remained intact after the release.

### Field trial on bait acceptability

**Figure 5.**
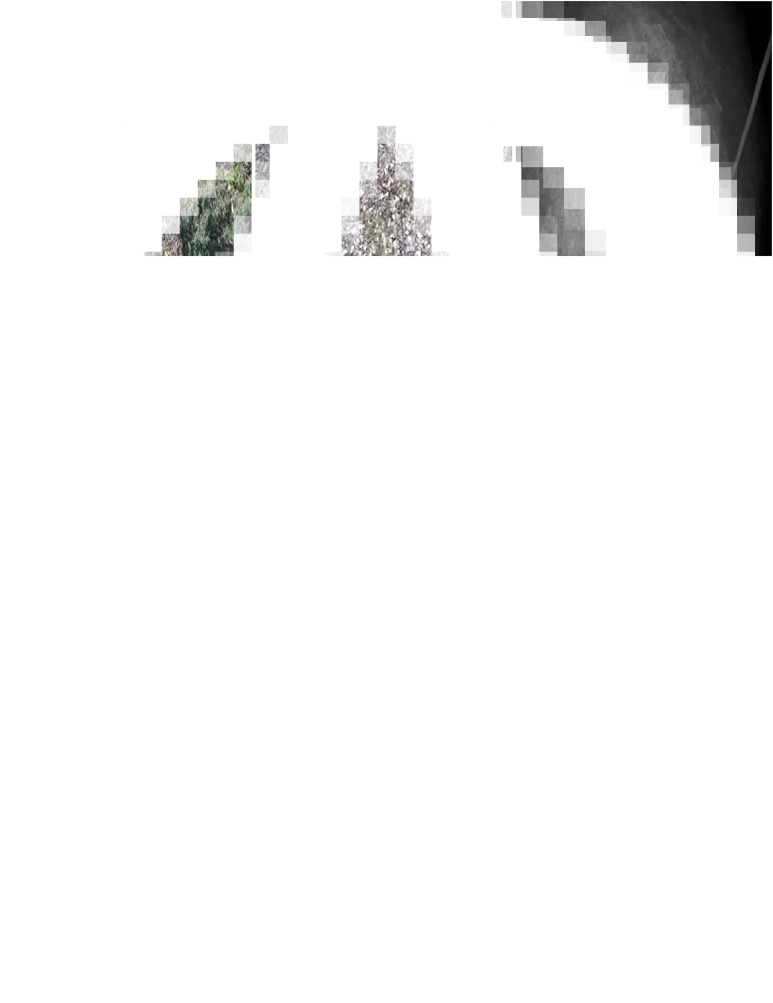
Images of the main consumers of the double PZQ-laced baits in the field captured by camera trap: not- owned dog (A), wild boar (B) and fox (C).

**Figure 6.**
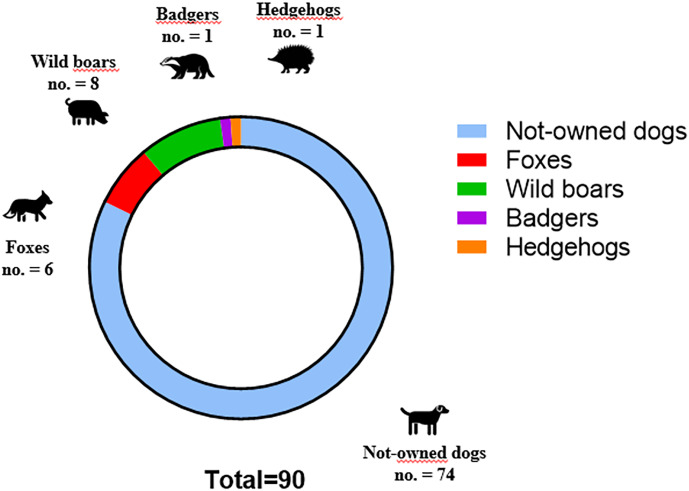
Number of occasions when baits were consumed by not-owned dogs and by different wildlife species.

A total of 90 double PZQ-laced baits were distributed in all 10 points of the pilot site. Among these, 72 (80%; Confidence Interval [CI]95% = 70.0–87.4%) were rapidly removed from the rural areas within 3 days. An analysis of the images showed that camera traps recorded 9 species frequenting baiting sites: not-owned dogs (no. movement images = 85), wild boars (no. = 18), foxes (no. = 14), hedgehogs (no. = 8), badgers (no. = 5), hares (no. = 3), martens (no. = 3), roe deer (no. = 2), wolves (no. = 1). Only 5 species fed on baits: most of the baits were eaten by not-owned dogs (74/90, 82.2%; CI95% = 72.4–89.2%) and the remaining by foxes (6/90, 6.7%; CI95% = 2.7–14.5%), wild boars (8/90, 8.9%; CI95% = 4.2–17.2) (Fig. 5), badgers (1/90, 1.1%; CI95% = 0.1–6.9) and hedgehogs (1/90, 1.1%; CI95% = 0.1–6.9) (Fig. 6). Martens, roe deer and wolves were not observed approaching the baits, while hares merely showed interest without consuming them. Furthermore, an analysis of the images showed that every not-owned dog did not consume the baits repeatedly in the same point of bait release.

## Discussion

This paper reports the results of a study aimed at assessing the best bait for deworming not-owned dogs to be included in effective control programmes in order to reduce the transmission of CE in endemic areas. Currently, prevention and control strategies for CE are based on an integrated approach, whose main actions can be listed as follows: regulation of slaughter activity and disposal of offal; prevention of dogs accessing offal; de-worming of shepherd/owned dogs (most frequently with PZQ); highly effective vaccination (with vaccine EG95) to prevent ovine CE; and public health education (Craig *et al*., 2017; Larrieu *et al*., 2017; Cringoli *et al*., 2021).

To date, only 3 control programmes implemented in countries and/or on islands (Iceland, New Zealand, Tasmanis, Kalkland Islands and Cyprus) have achieved eradication within 10–12 years, through specific policies focused on the control of CE in the dog definitive hosts (Craig *et al*., 2007). In other countries, despite the attempted control initiatives, CE remains a problem with a high infection rate, especially in the Mediterranean areas (Deplazes *et al*., 2017; Bosco *et al*., 2021; Cringoli *et al*., 2021). Most control programmes are based on the integration of different tasks as control strategies, including the anthelmintic treatment of shepherd dogs present on each farm (Craig *et al*., 2017). Deworming activities are usually performed with owned dogs tied to a chain or, by means of a purpose-built cage, known as ‘EchinoCage’, which made it possible to treat and analyse almost all the dogs present on the sheep farms (Cringoli *et al*., 2021), whereas the treatment of not-owned dogs has often been neglected.

Not-owned dogs are seen as a major contributor to the spread and persistence of CE in many countries, because they roam freely in grazing areas and prey on livestock offal, disseminating *Echinococcus* eggs into the environment. To date, no data are available concerning the environmental contamination with *Echinococcus* eggs in dogs (Deplazes *et al*., 2011). Hegglin and Deplazes (2013) estimated, instead, that, with respect to definitive host species, the contribution to environmental contamination with *Echinococcus multilocularis* eggs was of 400 to 6,600 eggs /m^2^, presenting a significant infection risk for humans and animals. Because of the importance of not-owned dogs in CE transmission, along with the extreme difficulties in treating them with anthelmintics (Kachani and Heath, 2014), new tools are needed to ensure that control programmes are implemented effectively.

In light of this background, the distribution of anthelmintic PZQ baits against wild and not-owned definitive hosts could be a good approach, leading to a significant reduction in CE prevalence. Baiting campaigns for alveolar echinococcosis control and oral rabies vaccination (ORV) (Comte *et al*., 2013; Umhang *et al*., 2019) have proven to be highly effective over the years, suggesting that the control of CE by treating not-owned dogs in extra-urban endemic areas is also feasible. The usefulness of PZQ-laced baits in the control of CE for the treatment of stray dogs has already been proven by Yu *et al*. (2017), but the use of baits containing PZQ has never been considered in Mediterranean countries as a new control action against CE. For this reason, there are several aspects to be considered in CE control when the use of medicated baits is included.

First, an important premise for a successful baiting campaign is the availability of a bait that is well accepted (highly palatable) by the target species under field conditions (Gibson *et al*., 2019). It is known that palatability is affected by several factors, such as physical aspect, taste and dosage form (Adenot and Abdelhakim, 2022). These factors are highly influenced by environmental exposure; in particular, rainfall, soil moisture, and temperature could play an important role in the longevity of baits (Allsop *et al*., 2017). Musto *et al*. (2022) have shown a high rate of degradation of the baits (loss of their characteristic scent and shape) used in central Italy for ORV campaigns in wild canids, which occurred after approximately 1 week due to weather conditions. These findings are in line with our study, where only the double PZQ-laced baits showed a better preservation up to 10 days under different climatic conditions, compared to the other 2 types of baits, which were found to be already broken or degenerated by the fifth day. This is most likely due to the presence of a double layer that avoids, for example, water penetration and thus increases the longevity of the bait. Indeed, the bait matrix (especially the ‘skin’ of the bait) greatly affects its integrity (Allsop *et al*., 2017).

Second, once the integrity of the bait was evaluated, their attractiveness and palatability over time were also assessed by preference testing (Thombre, 2004; Adenot and Abdelhakim, 2022) in order to find the best type of baits for the delivery on the field. Assessing the palatability of any proposed formulation on the target population prior to a baiting programme is an important step in predicting the effectiveness of that formulation for a particular population (Kappes *et al*., 2022). Although the baits tested in our study are all commercial products with a well-known high palatability, not all 3 baits showed the same attractiveness. The Milbemax^®^ Chewable tablets proved to be the least attractive in contrast to Petry *et al*. (2014), in which the dogs accepted the tablets well if taken directly from their owner's hand. In this study, it was observed that Milbemax^®^ Chewable tablets were not chosen voluntarily by dogs if distributed in a green space compared to other options (single and double PZQ-laced baits). This study found also that dogs' age, sex and breed had no significant impact on bait acceptance, as previously shown in other studies (Bender *et al*., 2017; Bonwitt *et al*., 2020).

Third, the last parameter tested was the mechanical resistance of the baits released from different altitudes. No damage was observed in all the double PZQ baits, distributed onto different surfaces and on different types of ground after drone release, up to heights of 25 m. A similar study was carried out during ORV campaigns, when the vaccine was distributed by helicopter onto solid ground at different altitudes and speeds, as reviewed by Mahl *et al*. (2014). The final aim of evaluating the integrity after release from different heights is to assess the feasibility of using drones for bait distribution, which might be useful in case of inaccessible areas (Yu *et al*., 2017). The custom drone used in this study was developed *ad hoc* for the delivery of medicated baits, aiming to improve deworming activities in the study area (Caputo *et al*., 2022) with advantages in terms of time, cost and manpower.

Since it is extremely difficult to recreate field conditions, the baits that had produced the best results during the preliminary trial (i.e. double PZQ-laced baits) were distributed manually in a grazing area, and their acceptance by not-owned dogs was investigated. Bait acceptance trials are an essential part of examining the feasibility of baiting campaigns (Estrada *et al*., 2001), and the best baiting strategy is characterized by high bait acceptance from target species and low bait acceptance from non-target species (Guthery *et al*., 1984). In this study, to investigate bait acceptance from target and non-target species camera traps were placed in strategic points and the acquired images were analysed. The evaluation of bait uptake using camera traps is a non-invasive control method that yields detailed data on bait competition between different species under different conditions (Hegglin *et al*., 2004). The camera traps photographed mostly not-owned dogs, and baits disappeared quickly from the baiting sites. The main competitors, i.e. non-target species, were wild boars and foxes, as also reported by Musto *et al*. (2022) in central Italy. However, it is important to point out that PZQ represents no risk for extra-urban wildlife, and the inadvertent treatment of non-target species presents minimal risk (Hegglin *et al*., 2004). The results obtained on the field underline that, despite high densities of wild animals, baits were mostly consumed by not-owned dogs, confirming the high attractiveness of double PZQ-laced baits for the target population. Furthermore, the continued removal of baits by not-owned dogs suggests that an anthelmintic baiting programme may be a viable tool for the management of *E. granulosus* among free-ranging dog populations in endemic rural areas. However, it is necessary to consider that to optimize the distribution of baits as well as the bait density, it should be taken in consideration the presence of target and non-target population in each area and adapt it accordingly, in order to significantly reduce the prevalence. In conclusion, while baiting for deworming not-owned dogs has been successfully tested in southern Italy, future perspectives will aim to validate it also in other countries of the Mediterranean area, in order to include these strategies in effective control programmes and reduce the transmission of CE in endemic areas.

## Data Availability

All reported data are available in this research article.
